# Functional immunophenotyping of children with critical status asthmaticus identifies differential gene expression responses in neutrophils exposed to a poly(I:C) stimulus

**DOI:** 10.1038/s41598-022-24261-y

**Published:** 2022-11-16

**Authors:** Jocelyn R. Grunwell, Milad G. Rad, Susan T. Stephenson, Ahmad F. Mohammad, Cydney Opolka, Anne M. Fitzpatrick, Rishikesan Kamaleswaran

**Affiliations:** 1 Children’s Healthcare of Atlanta, Egleston Hospital, Atlanta, GA USA; 2 Department of Pediatrics, Emory University School of Medicine, Atlanta, GA USA; 3 Department of Electrical and Computer Engineering, Georgia Institute of Technology, Atlanta, GA USA; 4 Department of Pediatrics, Division of Critical Care Medicine, Emory University School of Medicine, Children’s Healthcare of Atlanta at Egleston, 1405 Clifton Road NE, Atlanta, GA 30322 USA

**Keywords:** Asthma, Translational research, Transcriptomics, Neutrophils

## Abstract

The host immune response to a viral immune stimulus has not been examined in children during a life-threatening asthma attack. We determined whether we could identify clusters of children with critical asthma by functional immunophenotyping using an intracellular viral analog stimulus. We performed a single-center, prospective, observational cohort study of 43 children ages 6–17 years admitted to a pediatric intensive care unit for an asthma attack between July 2019 to February 2021. Neutrophils were isolated from children, stimulated overnight with LyoVec poly(I:C), and mRNA was analyzed using a targeted Nanostring immunology array. Network analysis of the differentially expressed transcripts for the paired LyoVec poly(I:C) samples was performed. We identified two clusters by functional immunophenotyping that differed by the Asthma Control Test score. Cluster 1 (*n* = 23) had a higher proportion of children with uncontrolled asthma in the four weeks prior to PICU admission compared with cluster 2 (*n* = 20). Pathways up-regulated in cluster 1 versus cluster 2 included chemokine receptor/chemokines, interleukin-10 (IL-10), IL-4, and IL-13 signaling. Larger validation studies and clinical phenotyping of children with critical asthma are needed to determine the predictive utility of these clusters in a larger clinical setting.

## Introduction

Despite recent advances in outpatient management of children with asthma, exacerbations remain common. In 2019, nearly half of all children with asthma experienced an asthma attack, 105 per 10,000 visited an emergency department, and 5 per 10,000 had a hospital inpatient stay^1^. While deaths from asthma have declined, hospitalizations are still prevalent and are driven by a small group of children who contribute to a substantial proportion (possibly up to 50%) of the total exacerbation burden^1,2^. Critical asthma is defined as an asthma attack severe enough to necessitate treatment in an intensive care unit^3–6^. These children with critical asthma likely have underlying intrinsic or biological risk^7^, but they remain poorly characterized.

Like outpatient asthma, critical asthma is a poorly characterized syndrome that is likely composed of multiple complex phenotypes. Whereas clustering and latent class analysis have been used to sub-classify outpatient asthma using clinical and biological variables^8–12^, similar approaches in children with critical asthma remain quite limited, in part due to challenges with performing phenotyping during the course of a hospital admission. We previously applied latent class analysis to a population of children with historical pediatric intensive care unit (PICU) admission and identified four latent classes that differed by demographic features, sensitization and type 2 inflammatory markers, prior exacerbation severity, health care use, and lung function^12^. However, there are no studies that define pediatric critical asthma phenotypes based on clinical history, triggers, and inflammatory markers while these children are hospitalized in the PICU.

Neutrophil-predominant asthma is described in children, albeit it less commonly than in adults^13^. Neutrophilic asthma is associated with pathogenic airway bacteria and poor response to corticosteroids compared to eosinophilic asthma^14–16^. Many children admitted to the PICU with status asthmaticus are triggered by respiratory viral infections, and neutrophils are first-line innate immune responders to viral components such as RNA and double-stranded DNA^17^. Neutrophils infected with virus activate immunostimulatory and antiviral gene expression programs via recognition of intracellular viral RNA by the pattern recognition receptors melanoma differentiation-associated gene 5 (MDA5) and retinoic acid-inducible gene I (RIG-I)^18^. In addition to antiviral transcriptomic stimulation, gene expression patterns in human neutrophils also change dramatically in response to bacteria exposure and by transmigration of circulating neutrophils into alveolar spaces^19–22^. For these reasons, we chose to assess the differential gene expression response of isolated blood neutrophils from children with critical asthma to an intracellular viral double-stranded RNA (dsRNA) analog, LyoVec low-molecular-weight (LMW) polyinosinic-polycytidylic acid (poly(I:C)).

Poly(I:C) has been used to evaluate the immune responsiveness to viral infections^23,24^. Low-molecular-weight (LMW) poly(I:C) complexed to the transfection agent LyoVec preferentially signals through the cytoplasmic retinoic acid-inducible gene-I (RIG-I) receptor inducing an inflammatory and anti-viral interferon-α (IFN-α) response that can be studied ex vivo^23^. Recently, LyoVec poly(I:C) was used for functional immunophenotyping of a cohort of critically ill children with influenza infections^23^. In that study, suppression of IFN-α production capacity was associated with increased disease severity, evidenced by an increased duration of mechanical ventilation and hospitalization and greater organ dysfunction. To date, the host immune response to a viral immune stimulus during a life-threatening asthma exacerbation has not been examined.

The objective of this study was therefore to determine whether functional immunophenotyping of critically ill children with status asthmaticus admitted to a PICU could be performed with ex vivo LyoVec poly(I:C) stimulation of isolated blood neutrophils. We hypothesized that clusters of children with life-threatening asthma could be identified by functional immunophenotyping and that these clusters would further be distinguished by other clinical features of asthma prior to hospitalization.

## Methods

### Patient cohort

This an ongoing single-center, prospective observational cohort study enrolling children ages 6–17 years admitted to the Emory University affiliated Children’s Healthcare of Atlanta, Egleston campus 36-bed PICU for status asthmaticus. The Emory University School of Medicine Institutional Review Board (IRB00110747) approved the study. Informed consent was obtained from a parent and/or legal guardian prior to enrollment, and all study procedures were in accord with the relevant guidelines and regulations in the Declaration of Helsinki. Participants were enrolled between July 29, 2019 and February 10, 2021. As this was an exploratory study, no power calculations were performed to determine sample size given the multidimensional outcomes assessed. Similarly, sufficient duration of study recruitment was not determined especially as the latter two years of study were conducted during the COVID-19 pandemic. Children are admitted to the PICU for status asthmaticus if they are receiving a third continuous nebulized albuterol treatment, are placed on non-invasive respiratory support delivered by high-flow nasal cannula or bilevel positive airway pressure or invasive mechanical ventilation, require an 80%/20% helium–oxygen (heliox) mixture, or require greater than or equal to 50% fraction of inspired oxygen by Venturi mask or positive pressure ventilation strategies. Children were excluded if they had chronic medical conditions requiring systemic corticosteroids or immunosuppressive medications such as those who have had a hematopoietic stem cell or solid organ transplant, an oncologic diagnosis, sickle cell anemia, or a rheumatologic diagnosis. Children with co-morbid respiratory disorders such as cystic fibrosis, pulmonary aspiration, gastroesophageal reflux requiring acid suppression medication and/or tube-feed dependence, bronchiectasis, congenital airway anomalies, bronchopulmonary dysplasia and/or a history of premature birth before 35-week gestation were excluded. Pregnant patients and those with a personal history of smoking were excluded.

### Characterization procedures

Medical history and treatments received were obtained from the Electronic Medical Record (EMR). Asthma symptoms in the four weeks prior to hospitalization were measured using the validated Childhood-Asthma Control Test (C-ACT) for those children between 6 and 11 years of age and the ACT for those children 12–17 years of age. A C-ACT or ACT score of 19 or less indicates uncontrolled asthma^25–27^.

### Blood collection and sample processing

Blood sample processing and neutrophil isolation has been previously described^28–30^. Blood was collected from participants at any point during their PICU admission through an existing peripheral intravenous catheter or central venous catheter into an ethylenediaminetetraacetic acid (EDTA) vacutainer tube and centrifuged at 400ΧG to separate cells from platelet-rich plasma. The majority of samples were collected within the first 48 h of hospitalization while the peripheral intravenous catheter still has blood return and the participants were receiving a 5-day burst of corticosteroids. Pelleted blood cells were resuspended in sterile phosphate buffered saline (PBS; ThermoFisher Scientific, Waltham, MA) with 2.5 mM EDTA up to the original whole blood volume. Neutrophils were purified by negative selection from PBS-EDTA washed whole blood using the EasySep Direct Human Neutrophil Isolation kit (StemCell Technologies, Cambridge, MA) according to the manufacturer’s protocol. There was > 99% neutrophil purity based on cytospin staining with Diff-Quik. Two million neutrophils were preserved in 1 mL of RNALater and stored at – 80 °C until RNA was isolated for baseline gene transcription analysis.

### Poly (I:C) stimulation

Four million neutrophils were resuspended in Roswell Park Memorial Institute (RPMI) medium (ThermoFisher Scientific, Waltham, MA) without serum. The neutrophil suspension was then split in half, and two million neutrophils were stimulated with 740 ng/ml low molecular weight (LMW) LyoVec poly(I:C) (hereafter referred to as poly(I:C), InvivoGen, San Diego, CA) resuspended in endotoxin-free water and the two million cells were incubated with vehicle solution for 20 h at 37 °C, 5% CO_2_ humidified incubator^23^. After centrifugation at 400ΧG, neutrophil pellets were saved in RNALater and frozen at – 80 °C until RNA was isolated for batch poly(I:C) gene transcription analysis.

### RNA preparation

RNA preparation was performed as previously described^28,29^. RNA was isolated from neutrophils using the Nucleospin RNA II kit with on-column genomic DNA digestion according to the manufacturer’s protocol (Takara, Mountain View, CA). RNA sizing quantification and quality control was performed in the Emory Integrated Genomics Core on an Agilent 2100 bioanalyzer using Pico and Nano Agilent kits and a Tecan optical density plate reader to measure the concentration of the RNA^28^. A low input RNA amplification kit was used. Only children with paired poly(I:C) gene expression data were used in the NanoString gene expression analysis.

### NanoString array

The Human Immunology v2 NanoString nCounter Gene Expression CodeSet was used (NanoString, Seattle, WA) and is comprised of 594 gene probes^29^. All NanoString-based measurements were conducted at the Emory University Integrated Genomics Core facility using an amplification step for low abundant RNA applied to all samples as previously described^29^. The Nanostring platform is forgiving of low abundant RNA with the use of an amplification step applied to all samples and is less prone to artifact from fragmented RNA compared with traditional sequencing approaches^29,31^. The datasets generated and analyzed during the current study are available in the Gene Expression Omnibus (GEO) repository at under the accession number GSE205151 using the persistent weblink https://www.ncbi.nlm.nih.gov/geo/query/acc.cgi?acc=GSE205151.

### Dimensionality reduction and cluster assignment of the paired poly(I:C) stimulated vs. unstimulated differentially expressed genes

After normalization of the gene expression data, dimensionality reduction and clustering was performed on the paired poly(I:C) stimulated vs. unstimulated samples using the Python uniform manifold approximation and projection (UMAP) umap-learn and scikit-learn package^32–34^. We used the UMAP python library for dimensionality reductions^35^. UMAP is a popular nonlinear graph-based dimensional reduction method routinely used for analysis of high-throughput sequencing data. A number of recent works have used the UMAP generated embedding within density-based clustering algorithms, such as k-means to achieve improved performance during unsupervised clustering^36,37^. We selected the optimal UMAP hyperparameters, namely neighbors and minimal distance based on the Pearson correlation and the cluster similarity score. We used the k-Means algorithm to assign clusters by separating samples into *n*-groups of equal variances by minimizing within-cluster sum of squares. The number of clusters were determined by using the Silhouette analysis.

### Pathway analysis by cluster assignment of the paired poly(I:C) stimulated vs. unstimulated differentially expressed genes

The Python Reactome package and the Kyoto Encyclopedia of Genes and Genomes (KEGG) were used to perform an over-representation gene network analysis using the differentially expressed genes in the paired poly(I:C) stimulated vs. unstimulated samples by cluster assignment^38–41^. Significant differentially expressed genes were determined using |Log_2_(fold-change)| greater than 1 and a false discovery rate (FDR) less than 0.05.

### Differential gene expression of baseline neutrophils by poly(I:C) cluster assignment

The differential gene expression of neutrophils stored immediately after negative selection were analyzed by clusters assigned in the paired poly(I:C) stimulated vs. unstimulated analysis using a Mann–Whitney *U* test.

### Statistical analysis

Differences in demographic, clinical, and biological features were assessed using Mann–Whitney *U* tests for non-parametric continuous variables and chi-square or Fisher’s exact test for categorical variables. Children with missing ACT or C-ACT measures were described in the overall cohort; however, these data were excluded from the asthma control outcome analysis. A *p*-value of less than 0.05 was considered significant.

## Results

### Cohort description

**Figure 1:**
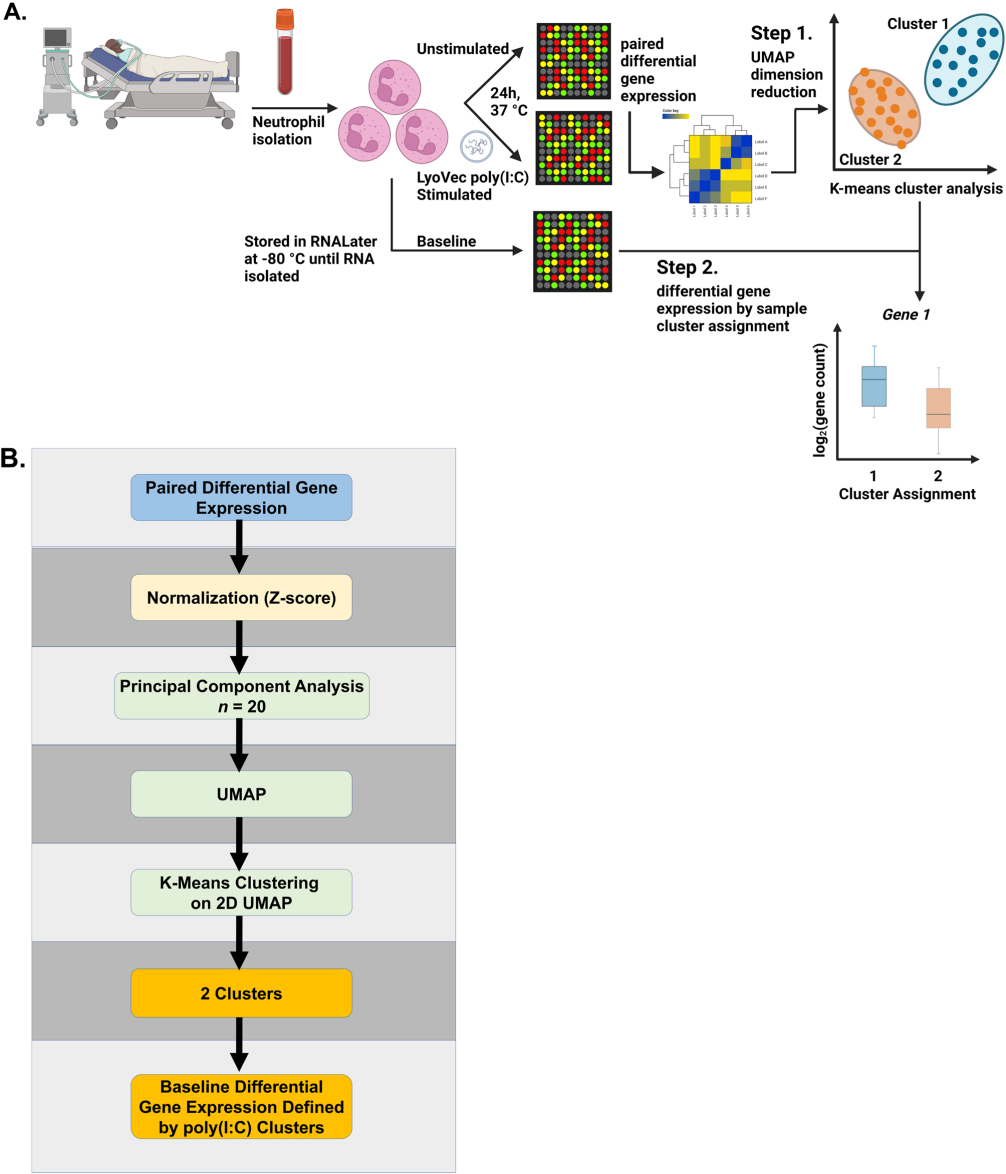
Overall study design sample processing (**A**). Neutrophils were isolated by negative magnetic bead selection from children ages 6–17 years admitted to the pediatric intensive care unit for an asthma exacerbation. Neutrophils (2 × 10^6^) were stimulated with low molecular weight (LMW) LyoVec poly(I:C) (740 n/ml) or vehicle solution for 20 h at 37 °C in a 5% CO_2_ humidified incubator. Following stimulation, neutrophils were isolated, resuspended in RNALater, and stored at – 80 °C until RNA was isolated for batch poly(I:C) gene transcription analysis using the Human Immunology v2 NanoString nCounter Gene Expression CodeSet. Following UMAP dimension reduction (Step 1), k-means clustering was used minimize within-cluster sum of squares and Silhouette analysis was performed to yield two clusters of differentially expressed genes. RNA was also isolated from neutrophils without any treatment (baseline). In Step 2, the differential gene expression of baseline neutrophils was analyzed using the two clusters defined by the poly(I:C) stimulated vs. unstimulated sample cluster analysis shown in Step 1. Data analysis workflow (**B**). Paired differential gene expression was determined using poly(I:C) stimulated vs. unstimulated samples. Differentially expressed genes were Z-score normalized. Principal component analysis was performed with 20 components, followed by uniform manifold approximation and projection (UMAP) dimensionality reduction. K-means clustering was performed on the two-dimensional UMAP to yield two clusters. The two poly(I:C)-stimulation defined clusters were used to analyze differentially expressed genes from the baseline neutrophils immediately isolated from patients with no stimulation. (**A**) was created with BioRender.com with a confirmation of publication and licensing rights.

**Figure 2:**
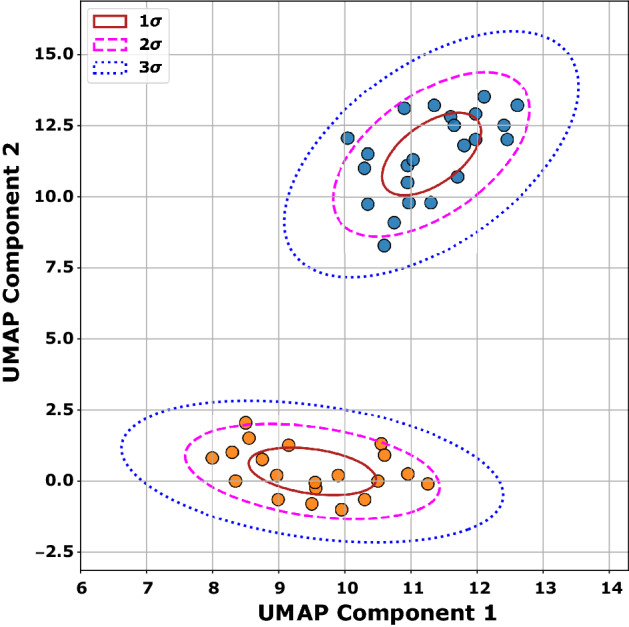
Uniform Manifold Approximation and Projection (UMAP) of component 1 versus component 2 showing two clusters of participants with differentially expressed genes following poly(I:C) stimulation. Cluster 1 (blue circles). Cluster 2 (orange circles). Ellipses indicate the boundaries of one standard deviation (σ) by a solid red line, two σ by a pink dashed line, and 3σ by a blue dotted line.

An overview of the study design and data analysis workflow is shown in Fig. 1A and B respectively. There were forty-three children with paired poly(I:C) gene expression data available for analysis. Children clustered into two groups based on the differential gene expression response to intracellular poly(I:C) (Fig. 2). Twenty-three children were assigned to cluster 1, and twenty children were assigned to cluster 2. The demographic and asthma history characteristics are summarized in Table 1. Children in cluster 2 were more likely to use a β-agonist daily and to have visited an emergency department or urgent care in that past year for an asthma exacerbation compared to children in cluster 1. Children in cluster 1 were more likely to be exposed to tobacco smoke and to have lower median C-ACT/ACT scores (15, IQR 14–19 vs. (18.5, IQR 15.5–22.8) indicative of uncontrolled asthma compared to children in cluster 2. There were no differences in emergency department and PICU treatment and laboratory study results by cluster (Supplementary Table 1). There were no differences in neutrophil count or immature band forms between the two clusters. There were no differences in duration of hospitalization or asthma exacerbations in the year following index hospitalization (Supplementary Table 2).

**Table 1:** Demographics and Asthma History of the Participants by Cluster.

Characteristic, n (%)	Cluster 1 *n* = 23	Cluster 2 *n* = 20	*p-*value
Age, year, median (Q1, Q3)	11.0 (9.0–3.5)	11.0 (7–14)	0.8
Sex: female/male	5 (22)/18 (78)	9 (45)/11 (55)	0.1
**Race/ethnicity**			
Black	21 (91)	18 (90)	0.3
White	2 (8.7)	0 (0)	
Other	0 (0)	2 (10)	
Hispanic or Latino	1 (4.3)	0 (0)	> 0.9
**Insurance**			
Private	4 (17)	5 (25)	0.7
Self-pay	0 (0)	1 (5)	
Government/medicaid	18 (78)	14 (70)	
Military/tricare	1 (4.3)	0 (0)	
History of asthma prior to PICU^a^	21 (95)^b^	15 (75)	0.087
Asthma control test	*n* = 20	*n* = 18	0.048
Median (Q1, Q3)	15 (14–19)	18.5 (16–23)	
**Home asthma medications**			
None	5 (22)	0 (0)	0.051
Albuterol or xopenex	14 (61)	13 (65)	0.8
Montelukast	12 (52)	9 (45)	0.6
ICS	6 (26)	5 (25)	> 0.9
ICS + LABA	7 (30)	8 (40)	0.5
Omalizumab	0 (0)	1 (5)	0.5
Daily oral corticosteroids	0 (0)	2 (10)	0.2
Daily use of β-agonist (at least 5/7 days)	7 (33)	10 (67)	0.048
**In past year, asthma resulted in**			
Emergency/urgent care visits	12 (57)	14 (93)	0.024
Hospitalized general ward	3 (14)	3 (20)	0.7
Hospitalized in PICU	4 (19)	3 (20)	> 0.9
**Past year, number of visits; median (Q1, Q3)**			
Emergency/urgent care visits	2 (1–4)	2 (1–2)	0.52
Hospitalized general ward	1.5 (0.8–2.2)	1 (1–9)	0.85
Hospitalized in PICU	1 (1–1)	1 (1–2)	0.24
**In lifetime, asthma resulted in**			
Hospitalized in PICU	11 (52)	9 (60)	0.7
Intubated for asthma	1 (4.8)	2 (13)	0.6
**Number of times in lifetime; median (Q1, Q3)**			
Hospitalized in PICU	1 (1–2)	3 (1–4)	0.12
Intubated for asthma	0.5 (0.25–0.75)	1 (1–1)	0.62
**Medical history**^**c**^			
None	4 (17)	1 (5)	0.4
Allergic rhinitis	6 (26)	4 (20)	0.7
Eczema	14 (61)	10 (50)	0.5
Sinusitis	1 (4.3)	0 (0)	> 0.9
**Family asthma history**^**c**^			
No one else has asthma	5 (22)	1 (5)	0.2
Father	8 (35)	4 (20)	0.3
Mother	9 (39)	7 (35)	0.8
Sibling	5 (22)	5 (25)	> 0.9
**Indoor exposures**^**c**^			
None	8 (35)	6 (30)	0.7
Tobacco smoke	10 (43)	3 (15)	0.043
Pets	9 (39)	4 (20)	0.2

^a^Pediatric intensive care unit; ^b^One Missing; ^c^More than one condition can be selected and frequency total may add up to more than 100%.

### Pathway analysis of differentially expressed genes by cluster assignment

**Figure 3:**
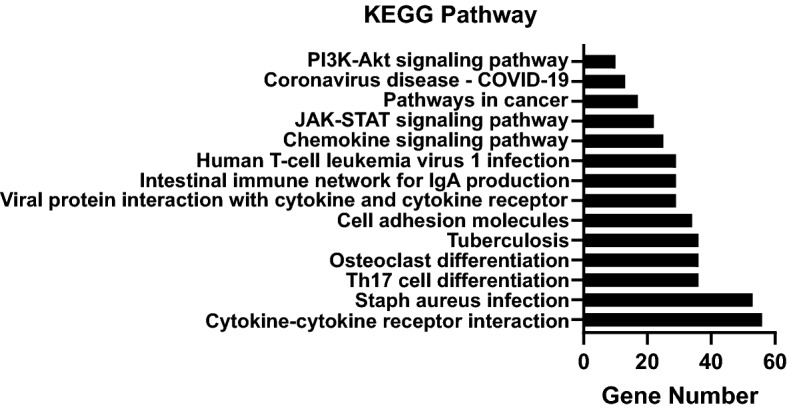
Bar graph of the top fourteen Kyoto Encyclopedia of Genes and Genomes (KEGG) pathways differentiating cluster 1 from cluster 2 by gene over-representation analysis (gene number) based on the differentially expressed genes following poly(I:C) stimulation.

There were 217 differentially expressed genes that distinguished cluster 1 from cluster 2 following poly(I:C) stimulation (Supplementary Fig. 1). The t-statistic and false discovery rate (FDR)-adjusted *p*-values for each of the genes in the paired poly(I:C) analysis are shown in Supplementary File 1. In general, differentially expressed genes in cluster 1 were upregulated compared to cluster 2. The differentially expressed genes were analyzed by over-representative analysis in Reactome pathway analysis and summarized in Table 2. Chemokine binding to receptors, interleukin-10 (IL-10), and the IL-4 and IL-13 signaling were the top three pathways of importance (Table 2). The top fourteen Kyoto Encyclopedia of Genes and Genomes (KEGG) pathways ranked by gene number are shown in Fig. 3. Cytokine-cytokine receptor interactions, *Staphylococcus aureus* infection, and Th17 cell differentiation were among the top 3 pathways in distinguishing the two clusters.

**Table 2:** Reactome pathways sorted by p-value for the 217 differentially expressed genes after LyoVec poly(I:C) stimulation by cluster.

Pathway name	Entities			Reactions	
	Found	Ratio	FDR^a^	Found	Ratio
Chemokine receptors bind chemokines	28/57	0.004	1.40e−14	16/19	0.001
Interleukin-10 signaling	23/86	0.006	1.40e−14	10/15	0.001
Interleukin-4 and interleukin-13 signaling	42/211	0.015	1.40e−14	28/47	0.003
Signaling by interleukins	91/643	0.044	1.40e−14	274/493	0.036
Cytokine signaling in immune system	116/1092	0.075	1.40e−14	347/708	0.052
Immune system	197/2681	0.184	1.40e−14	705/1623	0.12
Peptide ligand-binding receptors	30/203	0.014	1.40e−14	19/76	0.006
Innate immune system	75/1334	0.092	4.88e−14	249/710	0.053
Adaptive immune system	58/1004	0.069	6.05e−11	127/264	0.02
Class A/1 (rhodopsin-like receptors)	34/412	0.028	8.14e−12	24/159	0.012
Immunoregulatory interactions between a lymphoid and a non-lymphoid cell	28/316	0.022	1.31e−10	17/44	0.003
Interleukin-2 family signaling	11/47	0.003	5.49e−09	47/59	0.004
Complement cascade	17/156	0.011	3.53e−08	50/71	0.005
TNFR2 non-canonical NF-kB pathway	14/104	0.007	4.65e−08	19/43	0.003
GPCR ligand binding	34/606	0.042	1.25e−07	24/186	0.014
Costimulation by the CD28 family	12/97	0.007	1.02e−06	25/35	0.003
Regulation of Complement cascade	14/139	0.01	1.44e−06	31/42	0.003
Regulation of IFNA signaling	7/28	0.002	2.22e−06	4/5	3.70e−04
Interleukin-20 family signaling	7/29	0.002	2.79e−6	49/56	0.004
TNFs bind their physiological receptors	7/30	0.002	3.48e−06	8/13	9.62e−04
Regulation of IFNG signaling	5/16	0.001	2.26e−05	2/4	2.96e−04
Interferon signaling	22/394	0.027	2.49e−05	31/69	0.005
Terminal pathway of complement	4/8	5.50e-4	2.53e−05	5/5	3.70e−04
G alpha (i) signaling events	23/425	0.029	2.63e−05	6/74	0.005
Transcriptional regulation by RUNX1	17/261	0.018	3.33e−05	20/132	0.01

^a^False discovery rate.

### Baseline sample differential gene expression and pathway analysis by cluster assignment

**Figure 4:**
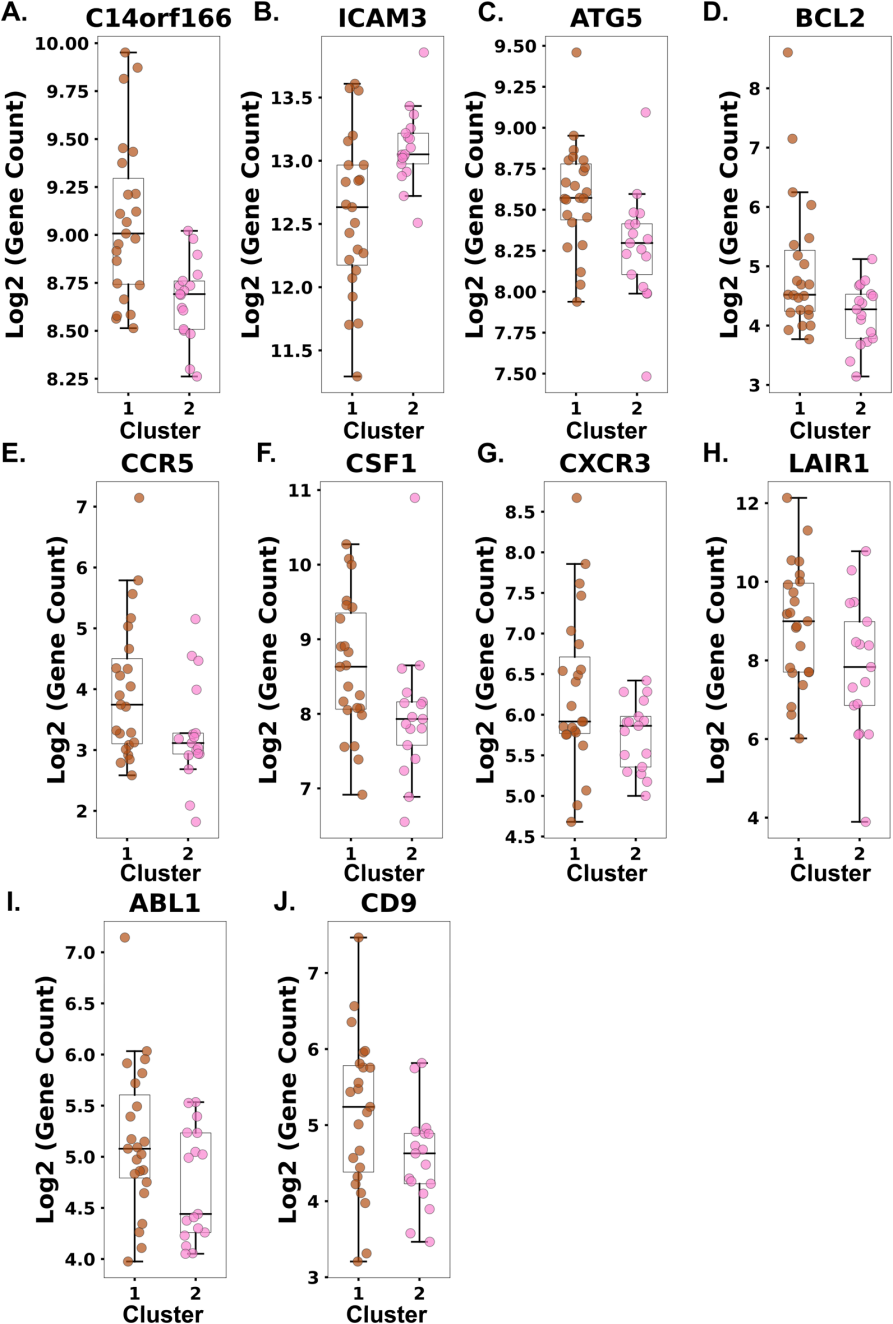
Box and whisker plots of the top ten differentially expressed genes by cluster from baseline (unstimulated) neutrophils. The middle line is the median, the bottom and top box edges are the 25th and 75th percentiles, respectively, and the whiskers are the minimum interval. (**A**) C14orf166, (**B**) Intercellular adhesion molecule 3 (ICAM3), (**C**) Autophagy-Related Gene 5 (ATG5), (**D**) B-cell lymphoma 2 (BCL2), (**E**) C–C chemokine receptor type 5 (CCR5), (**F**) Colony Stimulating Factor 1 (CSF1), (**G**) C–X–C Motif Chemokine Receptor 3 (CXCR3), (**H**) Leukocyte Associated Immunoglobulin Like Receptor 1 (LAIR1), (**I**) ABL Proto-Oncogene 1, Non-Receptor Tyrosine Kinase (ABL1), (**J**) CD9.

We next determined whether baseline gene expression differences were seen in the two clusters based on the cluster assignment following poly(I:C)-stimulation. The t-statistic and FDR-adjusted *p*-values for each of the genes in the baseline analysis by assigned cluster are shown in Supplementary File 2. There were ten genes that were differentially expressed (Fig. 4). These genes included: *C14orf166*, *ICAM3*, *ATG5*, *BCL2*, *CCR5*, *CSF1*, *CXCR3*, *LAIR1*, *ABL1*, and *CD9*. Reactome pathways from the list of ten differentially expressed genes included IL-10 signaling, cytokine signaling, IL-4 and IL-13 signaling, and the NLRP3 inflammasome (Table 3).

**Table 3:** Reactome pathways sorted by p-value for the 10 differentially expressed genes at baseline by cluster.

Pathway name	Entities			Reactions	
	Found	Ratio	FDR^a^	Found	Ratio
Interleukin-10 signaling	5/86	0.006	2.38e−06	2/15	0.001
Immune system	12/2684	0.188	2.62e−05	17/1623	0.12
Signaling by interleukins	7/643	0.045	6.16e−05	6/493	0.036
Cytokine signaling in immune system	7/1092	0.077	0.001	7/708	0.052
BH3-only proteins associate with and inactivate anti-apoptotic BCL-2 members	2/11	7.71e−04	0.001	3/4	2.95e−04
Estrogen-dependent nuclear events downstream of ESR-membrane signaling	2/ 29	0.002	0.007	1/12	8.84e−04
Interleukin-4 and interleukin-13 signaling	3/211	0.015	0.018	2/47	0.003
Innate immune system	6/1334	0.093	− 0.018	8/710	0.052
Chemokine receptors bind chemokines	2/57	0.004	0.018	2/19	0.001
Intrinsic pathway for apoptosis	2/64	0.004	0.02	4/62	0.005
Integrin cell surface interactions	2/86	0.006	0.032	1/55	0.004
Immunoregulatory interactions between a lymphoid and a non-lymphoid cell	3/316	0.022	0.032	2/44	0.003
The NLRP3 inflammasome	1/5	3.50e−04	0.032	1/4	2.95e−04
Extra-nuclear estrogen signaling	2/111	0.008	0.042	1/38	0.003
Acrosome reaction and sperm:oocyte membrane biding	1/6	4.20e−04	0.044	1/3	2.21e−04

^a^False discovery rate.

## Discussion

We performed an unsupervised cluster analysis of children with life-threatening asthma based on differential gene expression to an intracellular poly(I:C) viral analog stimulus. We identified two clusters of children distinguished by gene pathways involved in chemokine and cytokine signaling, Th17 cell differentiation, IL-10 signaling, and IL4/IL-13 signaling. Baseline characteristics of children did not differ between clusters; however, children in Cluster 1 had a lower median ACT score indicating worse asthma control compared to Cluster 2 patients. Despite Cluster 1 having worse asthma control compared to cluster 2, there was a larger proportion of emergency and urgent care visits in the year prior to index hospitalization and daily use of albuterol for the children in cluster 2. Ten differentially expressed genes differentiated the two clusters at baseline sample collection and included: IL-10 signaling, IL4/IL-13 signaling, estrogen receptor signaling, and the NLRP3 inflammasome.

Several adult and pediatric gene expression studies on peripheral blood mononuclear cells (PBMCs) have shown distinct gene expression profiles in eosinophil-predominant and neutrophil-predominant asthma. Neutrophil-predominant asthma is the most severe asthma phenotype with poor corticosteroid response^15^. Three clusters of pediatric asthma were defined using gene expression profiling of PBMCs distinguished by their inflammatory transcriptomic profiles; cluster 1 had the highest eosinophil count, cluster 2 had the lowest eosinophil and neutrophil counts, and cluster 3 had the highest neutrophil counts and the worse treatment control^15^. In their cohort of children, cluster 3 was defined by a T helper 1/T helper 17 (T_H_1/T_H_17) immune pathway response^15^. Interferon-γ (IFN-γ) is a hallmark of type 1 inflammation^17^, and 30% of adults with severe asthma have evidence of type 1 inflammation^42^. In our cohort, cluster 1 patients are characterized as having higher Th17 pathway response to an intracellular poly(I:C) stimulus compared with cluster 2 patients.

At baseline sample collection, cluster 1 patients had higher median levels of C–X–C receptor 3 (CXCR3) and C–C receptor 5 (CCR5) which promote chemotaxis of T_H_1 cells to sites of inflammation^43^. IFN-γ is produced by T_H_1 cells and leads to increased CXCL10 expression in a corticosteroid refractory manner. IFN-γ creates a positive feedback loop to not only augment the immune response to viral infection, but to also drive autoimmune pathology that is not responsive to corticosteroids. Inhibition of the CCR5 pathway has been shown to improve airway hyperreactivity with limited reduction in lung inflammation^43^.

The gene expression profile of the sputum of adolescents and adults with near-fatal asthma has been shown to have a higher airway transcriptome signature associated with epithelial cell differentiation, neurohumoral hemostasis, and histamine synthesis^44^. Whereas children in our study were enrolled during their life-threatening asthma episode and had neutrophils purified from peripheral blood samples, the prior study analyzed the transcriptomics of sputum samples from non-exacerbating adolescents and adults, some of whom had a history of near-fatal asthma^44^. Analysis of lung tissue from patients with fatal asthma has also shown increased expression of proinflammatory mediators such as granzymes and perforins released from CD8 + T cells and neutrophilic markers such as IL-8 and its receptor^45–49^.

Functional immune single-cell gene expression profiling of poly I:C-stimulated PBMCs from a biorepository of five adults with severe asthma and three healthy controls identified activation of pro-inflammatory and interferon pathways in the asthma group compared to healthy controls^50^. There were also a higher number of CD8 + T cells and CD8 + effector T cells in the severe asthmatic patients at baseline and a decrease following poly I:C stimulus compared to healthy controls in a complementary CyTOF analysis^50^. Despite the difference in cells stimulated and the difference in extracellular versus intracellular poly I:C substrate used for stimulation, we also found activation of the T _H_17/JAK/STAT and IFN-α/β/γ pathways.

We found several additional pathways of importance to asthma enriched in the baseline sample and stimulated transcriptomic differences in cluster 1 compared with cluster 2 including TNFα receptor binding, apoptosis, the NLRP3 inflammasome, and estrogen receptor signaling. Previous studies by Brown and Fitzpatrick have noted a subpopulation of children with moderate-to-severe asthma with persistently high TNFα who have poor asthma control despite high-dose corticosteroid treatment^51^. Activation of the NLRP3 inflammasome with production in the pro-inflammatory cytokine IL-1β and neutrophil extracellular trap (NET) formation drive steroid-resistant neutrophilic inflammation and airway hyperresponsiveness in allergic airway diseases^52–54^. A neutrophil-dominant, hard-to-treat, corticosteroid-resistant cluster of asthma in women has been identified; however, while we know that estrogen receptor signaling modulates allergic inflammation, we do not understand the interaction between glucocorticoid and estrogen receptors in corticosteroid-resistant asthma^55^.

To our knowledge, this is the first study to perform functional immunophenotyping in a cohort of children with critical asthma. However, there are several limitations to our study. First, we enrolled a small number of children from a single center, limiting the generalizability of the results. Given the critical nature of these children, comprehensive phenotyping including lung function measures was not performed. We also selected neutrophils from peripheral blood and performed analysis of bulk RNA making comparison to single-cell RNA-seq of PBMCs stimulated with poly(I:C) difficult. Additionally, all of the children in our cohort were receiving systemic corticosteroids at the time of blood sampling. The timing of blood sample collection was not standardized which could have introduced bias into the functional immune response measurement with children with earlier blood sampling having a gene expression response to intracellular poly(I:C) that was less impacted by corticosteroid treatment compared with later blood sampling. Many children in this study did not have viral respiratory panels sent due to the limited availability of nasal swabs and test reagents during the COVID-19 pandemic. Although viral respiratory infections may confound our cluster results, we did not detect differences in viral respiratory infection status between the two clusters in the limited number of samples where viral respiratory infection testing was performed. Although a higher proportion of children in Cluster 1 compared to Cluster 2 received heliox and isoflurane rescue therapies, the sample size is small and did not reach statistical significance. Validation of our results with a larger, well-phenotyped external cohort is needed to determine the reproducibility of the critical asthma response reported.

In summary, we defined two clusters of children with critical asthma by differential gene expression response to an intracellular poly(I:C) stimulus using a Nanostring platform and k-means clustering. Functional immunophenotyping may be an additional strategy to understand the heterogeneity of pediatric critical asthma. More detailed clinical phenotyping with lung function and endotyping with IgE, allergy testing, and plasma cytokines are needed to develop targeted, personalized therapies for children T_H_2 and non-T_H_2 life-threatening asthma.

## Supplementary Information

Supplementary Tables.

Supplementary Figures.

Supplementary Information 1.

Supplementary Information 2.

## Data Availability

The datasets generated and analyzed during the current study are available in the Gene Expression Omnibus (GEO) repository at under the accession number GSE205151 using the persistent weblink https://www.ncbi.nlm.nih.gov/geo/query/acc.cgi?acc=GSE205151.
