# Supplementary material for: Functional immunophenotyping of children with critical status asthmaticus identifies differential gene expression responses in neutrophils exposed to a poly(I:C) stimulus
Source: Sci Rep. 2022 Nov 16;12:19644. doi: 10.1038/s41598-022-24261-y (PMC9666940; doi:10.1038/s41598-022-24261-y)
Supplement: Supplementary file 1 — Supplementary Tables. [file 41598_2022_24261_MOESM1_ESM.docx]

**Functional Immunophenotyping of Children with Critical Status Asthmaticus Identifies Differential Gene Expression Responses in Neutrophils Exposed to a Poly(I:C) Stimulus**

Jocelyn R. Grunwell, MD, PhD^1,2^, Milad G. Rad, MS^3^, Susan T. Stephenson, PhD^2^, Ahmad F. Mohammad, BS^2^, Cydney Opolka, BS^1^, Anne M. Fitzpatrick, PhD^2^, and Rishikesan Kamaleswaran, PhD^2,3^

**SUPPLEMENTARY DATA**

**TABLE OF CONTENTS**

**Supplementary Table 1. Emergency Department and ICU Treatment and Laboratory Study Results**

**Supplementary Table 2. Duration of Hospitalization and Outcomes Following Hospitalization**

**Supplementary Figure 1.** Box and whisker plots of the significant differentially expressed genes defining the cluster from LyoVec poly(I:C)-stimulated versus vehicle control (unstimulated) neutrophils. The middle line is the median, the bottom and top box edges are the 25^th^ and 75^th^ percentiles, respectively, and the whiskers are the minimum interval.

**Supplementary File 1.** Ranked list of genes in the paired LyoVec poly(I:C) analysis with t-statistic and false discovery rate (FDR)-adjusted *p*-values.

**Supplementary File 2.** Ranked list of genes in the baseline analysis by cluster assignment with t-statistic and false discovery rate (FDR)-adjusted *p*-values.

**Supplementary Table 1. Emergency Department and ICU Treatment and Laboratory Study Results**

| Characteristic, n (%) | Cluster 1  *n* = 23 | Cluster 2  *n* = 20 | *p*-value |
| --- | --- | --- | --- |
| Type of Respiratory Support Prior to PICU  None  Regular nasal cannula  High-flow nasal cannula  CPAP or BiPAP  Intubation/mechanical ventilation | 0 (0)  1 (4.3)  10 (43)  10 (43)  2 (8.7) | 1 (5)  2 (10)  4 (20)  12 (60)  1 (5) | 0.4 |
| Medications Given Prior to ICU  Intermittent albuterol  Number, median (range)  Continuous albuterol  Magnesium sulfate (IV)  Corticosteroids  Inhaled  Oral  IV  Epinephrine (IM/SQ)  Terbutaline | 5 (22)  2.5 (2 - 6)  22 (96)  20 (87)  8 (35)  8 (35)  10 (43)  4 (17)  3 (13) | 2 (10)  1 (1 - 1)  20 (100)  17 (85)  6 (30)  7 (35)  7 (35)  4 (20)  1 (5) | 0.4  0.1  >0.9  >0.9  0.7  >0.9  0.6  >0.9  0.6 |
| Medication Given in ICU  Heliox (80/20)  Magnesium sulfate (IV)  Continuous albuterol  Terbutaline  Aminophylline  Isoflurane | 8 (35)  12 (52)  19 (83)  6 (26)  1 (4.3)  1 (4.3) | 3 (15)  9 (45)  16 (80)  3 (15)  1 (5)  0 (0) | 0.14  0.6  >0.9  0.5  >0.9  >0.9 |
| Ventilatory Support in ICU  No respiratory support  High-flow Nasal Cannula  Non-invasive positive pressure ventilation  Duration (days), median (range)  Invasive mechanical ventilation  Duration (days), median (range)  Isoflurane ventilator | 2 (8.7)  10 (43)  10 (43)  1 (0, 2)  2 (8.7)  6.5 (3 - 10)  1 (4.3) | 3 (15)  7 (35)  11 (55)  1 (0, 2)  1 (5)  1 (1 - 1)  0 (0) | 0.7  0.6  0.5  0.5  >0.9  0.7  >0.9 |
| CBC with Differential performed, n (%)  WBC Count (x10^9^ cells/L), median (range)  Absolute neutrophils, median (range)  Absolute eosinophils, median (range) | 6 (26)  14.1 (12.4 - 17.3)  11.3 (9.4 - 14.6)  0.35 (0.0 - 0.7) | 6 (30)  12.8 (9.2 - 15.9)  11.4 (8.2 - 20.8)  0.1 (0.0 - 0.6) | 0.3  0.48  0.94  0.17 |
| Baseline Blood Gas  ABG  CBG  VBG  pH, median (range)  pCO_2_ (mmHg), median (range)  pO_2_ (mmHg), median (range) | 2 (8.7)  2 (8.7)  11 (48)  7.33 (7.05 - 7.40)  42 (30 - 86)  47 (21 - 94) | 0 (0)  1 (5)  13 (65)  7.31 (7.04 - 7.36)  43 (29 - 99)  43 (20 - 117) | 0.5  >0.9  0.3  0.14  0.74  0.62 |
| Viral Respiratory Panel^a^  Not performed  No pathogen detected  Rhinovirus/Enterovirus  Mycoplasma pneumoniae | 15 (65)  2 (8.7)  6 (26)  0 (0) | 15 (75)  3 (15)  2 (10)  1 (5) | 0.5  0.7  0.3  0.5 |

^a^Detects coronavirus 229E, HKU1, OC43, NL63; SARS-CoV2 is not detected by this test

**Supplementary Table 2. Duration of Hospitalization and Outcomes Following Hospitalization**

| Characteristic, n (%) | Cluster 1  *n* = 23 | Cluster 2  *n* = 20 | *p-*value |
| --- | --- | --- | --- |
| Length of Stay, median (Q1, Q3)  ICU (days)  Hospital (days) | 2 (1.5 - 2.5)  2 (2 - 3) | 2 (1 - 3)  2 (1 - 3) | 0.51  0.13 |
| Discharged to  Inpatient Rehabilitation  Home | 1 (4.3)  22 (96) | 0 (0)  20 (100) | >0.9  >0.9 |
| Discharge Medications  Albuterol or Xopenex  Montelukast  ICS  ICS + LABA  Omalizumab Candidate  Oral corticosteroid Burst  Dexamethasone  Prednisolone  Prednisone  Daily oral corticosteroids | 23 (100)  14 (61)  12 (52)  10 (43)  0 (0)  17 (74)  1 (5.9)  7 (41)  9 (53)  7 (30) | 20 (100)  9 (45)  8 (40)  10 (50)  0 (0)  10 (50)  0 (0)  3 (30)  7 (70)  12 (60) | -  0.3  0.4  0.7  -  0.11  0.052 |
| Primary Outcome within 1 year of index hospitalization  Any medical visit for asthma exacerbation  Multiple Exacerbations | 12 (52)  4 (17) | 10 (50)  3 (15) | 0.9  >0.9 |
| Secondary Outcomes within 1 year of index hospitalization  Time to first asthma exacerbation (days)  Number of Exacerbations, median (range)  Location of visit  Urgent Care/Emergency Department  Asthma/Allergy Clinic  Number of Asthma/Allergy Clinic Visits, median (range)  Number of total visits, median (range)  Hospitalized (Yes)  Number of inpatient admissions, median (Q1-Q3)  General Ward  ICU | 26 (14 - 117)  2.5 (2 - 5)  7 (30)  13 (57)  2 (0 - 4)  0 (0 - 1)  3 (13)  2.5 (1.25 - 3.75)  1 (0.75 - 1) | 72 (31 - 168)  4 (2 - 13)  6 (30)  9 (45)  1.5 (0 5)  0 (0 - 1)  3 (15)  0.5 (0 - 1)  0.5 (0 - 1) | 0.45  0.58  >0.9  0.46  0.5  0.9  >0.9  >0.9  0.81 |
