# Supplementary figures and images for: Functional immunophenotyping of children with critical status asthmaticus identifies differential gene expression responses in neutrophils exposed to a poly(I:C) stimulus
Source: Sci Rep. 2022 Nov 16;12:19644. doi: 10.1038/s41598-022-24261-y (PMC9666940; doi:10.1038/s41598-022-24261-y)

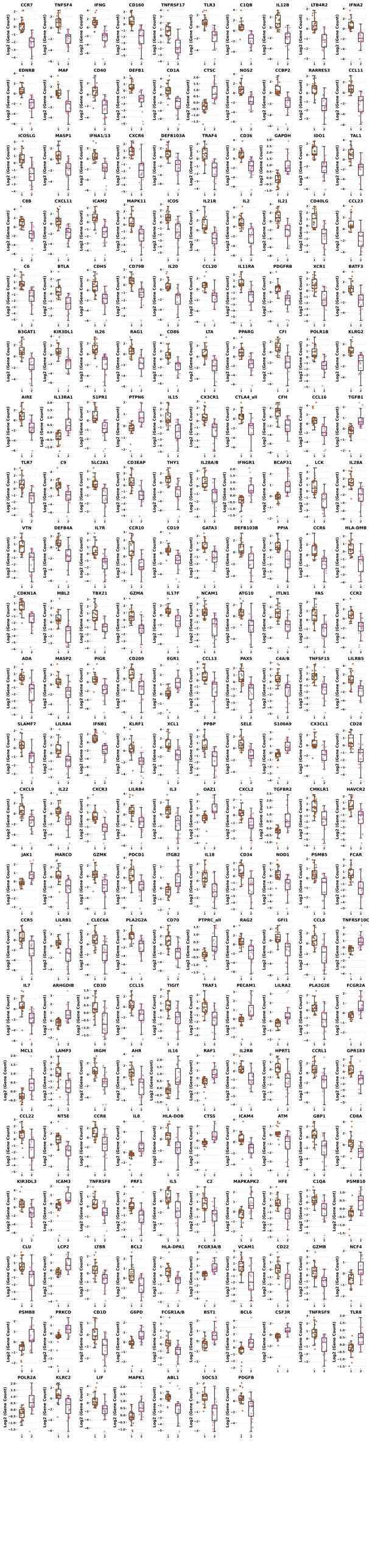

Supplement: Supplementary file 2 — Supplementary Figures. [file 41598_2022_24261_MOESM2_ESM.pdf]
